# The relationship between autonomy, relatedness, and students' transformative experience: a cross-cultural study

**DOI:** 10.3389/fpsyg.2025.1527510

**Published:** 2025-07-09

**Authors:** Yousef Alardhi, Kevin Pugh

**Affiliations:** ^1^Department of Educational Psychology, College of Education, Kuwait University, Kuwait City, Kuwait; ^2^School of Psychological Sciences, University of Northern Colorado, Greeley, CO, United States

**Keywords:** transformative experience, autonomy, relatedness, self-determination theory, pre-servce teachers

## Abstract

Transformative experience is a deep-engagement outcome that involves using curricular content to see and experience the world in meaningful new ways in one's everyday life. Researcher have investigated individual factors predictive of transformative experience. We built on this research by investigating perceived autonomy and relatedness support as potential predictors of transformative experience and considering cross-cultural effects. Pre-service teachers from both the United States and Kuwait comprised the sample (*n* = 353). Perceived autonomy and relatedness support, the interaction between perceived autonomy and relatedness support, and the three-way interaction between perceived autonomy support, perceived relatedness support, and nationality were significant predictors of transformative experience controlling for students' year in school and gender. Moderation analyses revealed that a baseline level of perceived relatedness support was needed for the relationship between perceived autonomy support and transformative experience to manifest and this baseline was higher for Kuwaiti students. Further, for U.S. but not Kuwaiti students, a baseline level of perceived autonomy support was needed for the relationship between perceived relatedness support and transformative experience to manifest. Overall, perceived autonomy and relatedness support were important predictors of transformative experience for both nationalities, but perceived autonomy support was relatively more important for U.S. students and perceived relatedness support for Kuwaiti students.

## Introduction

Current definitions of teacher quality are less concerned with character attributes or technical skill and more focused on instructors' capacity to engage students in demanding, meaningful activities that promote educational experiences for all students (Mitchell et al., 2001). One aspect of this modern vision of teacher quality is a concern with making learning transformative; that is, fostering experiences in which students make deep connection to the content and meaningfully apply it in their everyday lives. We believe that for teachers to be proficient in fostering transformative learning experiences, they need to undergo such experiences in their own pre-service teacher experience. To a significant degree, teachers develop pedagogical principles that will drive their future efforts based on their undergraduate student experiences (Kennedy, 1999; Cochran-Smith and Zeichner, 2009). Consequently, it is critical that pre-service teachers undergo educational experiences that are transformative. A first step to achieving this goal is to identify factors associated with undergoing transformative experiences for pre-service teachers.

One means of conceptualizing transformative learning is through the construct of *transformative experience*. Transformative experiences occur when students engage in meaningful learning that allows them to view the world in a new and meaningful way in their everyday experience (Pugh, 2020). Transformative experience is a deep form of behavioral, cognitive, and affective engagement because it requires students to go beyond classroom engagement, apply their learning in their out-of-school lives, and find value in doing so (Pugh, 2011; Pugh et al., 2020). Researchers have investigated factors associated with undergoing transformative experiences (for a review, see Pugh et al., 2023a). However, research targeting pre-service teachers is lacking. In addition, a gap in the literature is an investigation of the potential importance of the perceived basic psychological needs of autonomy, competence, and relatedness (Ryan and Deci, 2017, 2020). These perceived needs predict intrinsic motivation (Ryan and Deci, 2017), which shares a focus on self-directed action with transformative experience. Hence, these perceived needs may also predict transformative experiences.

In our research, we were particularly interested in students perceived psychological need support; that is, students' perception of the degree to which their environment (e.g., educational setting) supports their psychological needs.^1^ Understanding the relation between need support and transformative experience has implications for identifying environments that may foster transformative experiences. Accordingly, we investigated perceived autonomy and relatedness support as predictors of transformative experience among pre-service teachers. Perceived competence support was not investigated in the current study because we desired to do a deeper analysis of the potential interaction of perceived autonomy and relatedness support in predicting transformative experience.

Furthermore, we chose to investigate potential differences between U.S. and Kuwaiti students regarding the predictive relationships and interactions between perceived autonomy support, relatedness support, and transformative experience. Cross-cultural research on transformative experience is absent and it is possible that perceived autonomy and relatedness support interact with culture in predicting transformative experience. We chose U.S. and Kuwaiti samples as the U.S. represents an individualistic culture and Kuwait represents a collectivist culture.

## Literature review

### Transformative experience theory

Transformative experience theory draws on Dewey's (1938; 1980/1934) theories of aesthetic and educative experience to conceptualize meaningful learning experience (Pugh, 2002; Pugh et al., 2020; Wong et al., 2001). Dewey argued that aesthetic experiences were transformative in that they expanded perception and value in everyday experience (Dewey, 1933). Moreover, he believed education can and should have a similar focus. That is, education should enrich, expand, and transform everyday experiences (Dewey, 1938). However, Dewey (1938, 1990/1902) lamented that the curriculum had become an aim unto itself with little consideration for its experiential effects. Arguably, this concern still applies. How learning might enrich and expand students' everyday experience is often an afterthought, if considered at all.

To address this concern, Pugh and colleagues (e.g., Pugh, 2002; Pugh and Girod, 2007; Wong et al., 2001) sought to conceptualize Dewey's view of aesthetic, transformative experience more precisely such that it could be researched empirically. Toward this end, Pugh (2011) defined a transformative experience as a “learning episode in which a student acts on the subject matter by using it in everyday experience to perceive some aspect of the world and finds meaning in doing so more fully” (p. 111). Further, he proposed three defining characteristics derived from Dewey's theory of aesthetic and educative experience: motivated use, expansion of perception, and experiential value. Motivated use refers to the use of educational material in non-required circumstances, especially outside of the given class. Expansion of perception refers to the ability of students to view the world and their everyday experiences through the lens of curricular concepts. Experiential value refers to developing a deeper appreciation for the curricular concepts that expand perception and for aspects of the world that are re-seen through the lens of the curriculur concepts. For example, after learning about learned helplessness in an educational psychology course, a pre-service teacher may choose to think about this content when interacting with friends and observing classrooms (motivated use), come to perceive friends and students through the lens of different attribution patterns (expansion of perception), and come to value the way this content enriches understanding of people and their actions (experiential value).

Researchers have clarified the meaning of transformative experience (Pugh, 2004; Pugh et al., 2017), validated a measure of transformative experience (Koskey et al., 2018), identified learning outcomes positively associated with transformative experience (e.g., enduring learning, Girod et al., 2010; Pugh, 2002; transfer of learning, Pugh et al., 2010; conceptual change, Alongi et al., 2016; Heddy and Sinatra, 2013; domain interest, Heddy et al., 2023; Heddy and Sinatra, 2013; academic and career choice, Manzanares and Pugh, 2025; Pugh et al., 2021), and investigated pedagogical strategies effective at fostering transformative experiences (see Pugh, 2020 for a review). One additional line of research has focused on identifying factors predictive of transformative experiences. This research is particularly relevant to the current research and is reviewed below.

### Factors predictive of transformative experience

Initial case study research suggested students were more likely to undergo transformative experience when they identified with the domain (Girod and Wong, 2002; Pugh, 2004). Building on these findings, researchers found science identity to be a significant predictor of transformative experience in survey research (Pugh et al., 2010). Further, the researchers found the relationship between science identity and transformative experience to be mediated by students' goal orientation. Students with a stronger science identity were more likely to adopt a mastery goal orientation (i.e., a focus on learning and developing competence; Ames, 1992), which in turn predicted transformative experience.

In addition, researchers found domain interest to be an important predictor of transformative experience (Heddy et al., 2023; Pugh et al., 2021, 2023b). Pugh et al. (2019) found maintained situational interest and openness to experience to be significant predictors of transformative experience, with the relationship between openness and transformative experience being mediated by maintained situational interest. Relatedly, Pugh et al. (2019) found that students who reported high levels of positive emotions and task value and low levels of negative emotions, anxiety, and perceived cost had the highest transformative experience. Students' perceptions of their teachers have also been identified as factors related to transformative experience. Specifically, Pugh et al. (2023b) found that students' perceived connection to their teachers and their perception of their teachers' passion for the content were significant predictors of transformative experience, even controlling for domain interest and efficacy.

One potentially fruitful area of future research is investigating perceived basic psychological need support as a predictor of transformative experience. Within self-determination theory, autonomy, competence, and relatedness have been identified as basic psychological needs (Ryan and Deci, 2000, 2017). Moreover, perceived satisfaction and support of these needs is predictive of intrinsic motivation (Ryan and Deci, 2000, 2017; Niemiec and Ryan, 2009). Because intrinsic motivation shares a focus on self-directed action with transformative experience, it is possible that these basic psychological needs predict transformative experience. In the current study, we considered how perceived autonomy support and perceived relatedness support relate to transformative experience. In addition, we investigated how they might interact in predicting transformative experience and function differently across cultures.

#### Perceived autonomy support as a potential predictor of transformative experience

The need for autonomy refers to a feeling of ownership and free will in one's actions (Deci and Ryan, 2000). Compared to a controlling context, an autonomy supportive context is positively associated with greater autonomous motivation, particularly intrinsic motivation (Ryan and Deci, 2017). In addition, an autonomy supportive context is associated with greater interest (Gillet et al., 2012), task value (Patall et al., 2013), cognitive, behavioral, and emotional engagement (Gutiérrez and Tomás, 2019; Hospel and Galand, 2016; Jang et al., 2010; Reeve et al., 2004; Finn and Zimmer, 2012; Benita and Matos, 2021), mastery goal orientation (Benita and Matos, 2021), self-efficacy (Gutiérrez and Tomás, 2019), and academic achievement (Gutiérrez and Tomás, 2019). An autonomy supportive context is also associated with lower dropout intentions and anxiety (Black and Deci, 2000), lower academic stress (Zheng et al., 2020), and lower grade-focused performance goals (Black and Deci, 2000). Given that some of these outcomes are also factors predictive of transformative experience (e.g., interest, positive emotions, mastery goal orientation), it is likely that perceived autonomy support predicts transformative experience. Further, as stated previously, intrinsic motivation and transformative experience share a common focus on self-directed action and, thus, factors associated with intrinsic motivation may also be associated with transformative experience.

#### Perceived relatedness support as a potential predictor of transformative experience

Relatedness is a second fundamental psychological need (Baumeister and Leary, 1995; Maslow, 1943; Ryan and Deci, 2017). Ryan and Deci (2020) defined relatedness as a feeling of connection and care. Relatedness is also commonly defined as a student's feeling of being valued, included, and accepted (Goodenow and Grady, 1993; Maunder, 2018; Masika and Jones, 2016; Meehan and Howells, 2019; Slaten et al., 2016). Previous research demonstrated that a higher sense of relatedness is associated with autonomous motivation, particularly intrinsic motivation (Ryan and Deci, 2017; Freeman et al., 2007). As with autonomy, relatedness is also positively associated with several adaptive outcomes including retention (Pedler et al., 2022), cognitive, behavioral, and emotional engagement (Finn and Zimmer, 2012; Gillen-O'Neel, 2019; Gopalan and Brady, 2020; Wilson et al., 2015; Zumbrunn et al., 2014), enjoyment (Pedler et al., 2022), self-efficacy and positive task value (Freeman et al., 2007), lower stress (Civitci, 2015), and better academic adjustment, achievement, and aspirations (Strayhorn, 2018). Given the connection between these outcomes and predictors of transformative experience and the ties between intrinsic motivation and transformative experience, we believe it is likely that perceived relatedness support will predict transformative experience.

#### Potential interaction between perceived autonomy and relatedness support in predicting transformative experience

As pointed out by Kluwer et al. (2020), it may appear that autonomy and relatedness are negatively related since autonomy refers to self-directed choice and decision ownership while relatedness refers to connection and closeness; achieving one may appear to undermine the other. However, Kluwer et al. (2020) explained that autonomy should not be confused with independence or isolation from others. Autonomy in this context means the experience of having choice, agency, and initiative while yet feeling connected to others. Moreover, Deci and Ryan (2000, p. 253) noted that this connection between autonomy and relatedness is natural to humans under ideal conditions:

Thus, much of the rich fabric of the human psyche is founded upon the interplay of the deep adaptive tendencies toward autonomy (individual integration) and relatedness (integration of the individual into a larger social whole) that are part of our archaic heritage and will, under optimal circumstances, be complementary but can, under less optimal circumstances, become antagonistic.

In line with self-determination theory, Kluwer et al. (2020) found in three studies that autonomy and relatedness positively interact in predicting optimal relationship functioning. Relatedness was associated with relationship maintenance behavior only when participants reported high levels of autonomy. Consequently, it is possible that perceived autonomy and relatedness support will interact when predicting transformative experience. It is anticipated that a base level of perceived autonomy support may be necessary for a relationship between perceived relatedness support and transformative experience to occur and vice versa.

#### Cross-cultural comparison of perceived autonomy and relatedness support as predictors of transformative experience

One significant gap in transformative experience theory is consideration of how transformative experience and factors related to transformative experience may vary across populations and contexts. Only a few studies have addressed this issue. In the context of university-level geoscience education, Pugh et al. (2019, 2021) investigated gender differences related to transformative experience. Pugh et al. (2019) found that female students reported slightly lower levels of transformative experience than male students. Further, Pugh et al. (2021) found that transformative experience contributed to explaining confidence in a geoscience major and intent to pursue a geoscience career for female but not male students. Goldman et al. (2024) found no difference between first-generation and non-first-generation students in levels of transformative experience or predictive relationships in terms of variables predicting transformative experience.

To date, no researchers have investigated transformative experience in non-U.S. settings and no cross-cultural research has been conducted. This is a significant limitation in the existing research as it is problematic to assume that transformative experience will have the same prevalence, functioning, and applicability across cultures. Given that gender differences were found in prior research (Pugh et al., 2019, 2021), it is probably that cultural differences exist. Accordingly, there is need for cross-cultural research on transformative experience.

Cross-cultural research associated with self-determination theory provides context for considering the relevance of cross-cultural research to transformative experience theory. According to Ryan and Deci (2002), basic psychological needs are innate requirements as opposed to acquired motivations; consequently, these needs exist across all cultures. However, other researchers argue that these needs are not universal and dependent on culture (Iyengar and Lepper, 1999; Markus and Kitayama, 2003). Markus and Kitayama (2003) argued that the concept of autonomy is thought to hold importance for individuals' wellbeing in Western civilization, which emphasizes individualism, but has little value in Eastern collectivist cultures that value a group's needs. In addition, due to the nature of Eastern cultures, their major psychological need might be relatedness (Heine et al., 1999).

The existing research lends some support for each of these positions. First, numerous studies support the universal role of the three basic psychological needs (e.g., Chirkov and Ryan, 2001; Magson et al., 2024; Oga-Baldwin et al., 2017) and need-supportive teaching has been found to be universally important for socio-emotional learning across cultures (Wang et al., 2024). However, researchers also found cultural differences in people's views of autonomy and variance in the importance of different needs in different cultures (e.g., Cheng et al., 2016; Magson et al., 2024). Ryan and Deci (2020) proposed a synthesis view in which both the universality and the culturally specific manifestation of the basic psychological needs are acknowledged: “Specifically, SDT makes etic claims concerning the universal importance of its basic psychological needs for autonomy, competence, and relatedness, yet it also recognizes emic variations in the salience, meaning and dynamics of needs between cultures” (p. 5). While existing research acknowledges both the universal importance and culturally variable manifestations of basic psychological needs (Ryan and Deci, 2020), it is crucial to avoid overly simplistic interpretations that directly link autonomy to individualism and relatedness to collectivism. Such a binary view overlooks the complexity of cultural influences, where autonomy and relatedness can be valued differently even within the same cultural context (Vignoles et al., 2016). We adopted a culturally sensitive approach that recognizes this complexity, investigating how autonomy and relatedness support differ between U.S. and Kuwaiti students without assuming a direct mapping of these needs to cultural categories.

In line with this existing research, we anticipate that perceived autonomy and relatedness support will be important psychological needs across cultures; however, their salience in explaining engagement in transformative experience may vary. Consequently, we feel a need to investigate whether the relationship between transformative experience and perceived basic psychological needs support varies by culture. In the current study, we investigated potential differences between collectivist and individualistic cultures.

In collectivistic civilizations, the self is interconnected, while it is autonomous in individualistic cultures (Markus and Kitayama, 1991). Additionally, in collectivist societies, communal aims take precedence over individual ones, but in individualistic cultures, individual goals are prioritized (Triandis, 1995). In collectivist societies, norms and obligations govern people's conduct, but in individualistic cultures, personal choice and a person's rights drive people's behavior (Triandis and Gelfand, 2012).

Kuwait was selected as a cross-cultural comparison site as it is an understudied population and represents Eastern collectivist society as the score of individualism is low (25) (Hofstede Insights, 2023). The United States was selected as a representative Western individualistic society where the score of individualism was found to be high (91) (Hofstede Insights, 2023). Given these differences between the U.S. and Kuwait, it is possible that perceived autonomy and relatedness support differ in their salience to these two populations of students and differ in the degree to which they help explain the undergoing of transformative experiences. However, research is needed.

### Pre-service teachers' transformative engagement

Currently, there is a lack of research on pre-service teachers' transformative experiences. Younis (2021) found that transformative experiences were high among pre-service teachers participating in a photovoice intervention, but research is lacking on factors explaining pre-service teachers' differing levels of transformative experience. Nevertheless, there is research on factors predictive of the related outcomes of pre-service teacher engagement and intrinsic motivation. In a multicultural study, Kaplan and Madjar (2017) found that supporting pre-service teachers' basic psychological needs was positively associated with autonomous motivation. The authors also showed that autonomous motivation was positively associated with self-accomplishment, engagement, and self-exploration. Mediated by motivational climate, supporting autonomy was associated with higher engagement for pre-service teachers during COVID-19 lockdown (López-García et al., 2022).

## Current study

The purpose of the current research was to understand the potential relationship between perceived autonomy and relatedness support and transformative experience among pre-service teachers. In our investigation, we examined the relationships while controlling for students' year in school and gender. The purpose of exploring these relationships under these covariates is rooted in the limited existing knowledge about these dynamics, thereby contributing valuable insights to the field. In addition, we investigated potential interaction effects between perceived autonomy and relatedness support and whether such interaction effects differed between U.S. and Kuwaiti pre-service teachers.

### Research questions

1. Controlling for school year and gender, do perceived autonomy and relatedness support predict transformative experience?
2. Hypothesis: Based on research finding that perceived autonomy and relatedness support are key factors in predicting intrinsic motivation (Ryan and Deci, 2017), we hypothesized these factors would also be positive predictors of transformative experience.
3. Controlling for school year and gender, is there an interaction between perceived autonomy and relatedness support in predicting transformative experience?
4. Hypothesis: Based on prior research findings that autonomy and relatedness positively interact when predicting desirable outcomes (Deci and Ryan, 2000; Kluwer et al., 2020), we hypothesized that they would positively interact in predicting transformative experience.
5. Controlling for school year and gender, is there a three-way interaction between perceived autonomy support, relatedness support, and nationality (U.S. vs. Kuwait) in predicting transformative experience?
6. Hypothesis: Based on prior research finding differences in the importance of autonomy and relatedness in collectivist vs. individualistic countries (e.g., Magson et al., 2024), we predicted a significant three-way interaction.
7. 3a. Assuming a three-way interaction is found, how do moderation effects help us understand the three-way interaction?

Hypothesis: Based on claims by Ryan and Deci (2020) about the universality and context specific manifestation of basic psychological needs, we hypothesized that perceived autonomy and relatedness support would be significant predictors of transformative experiences for both nationalities; however, autonomy would be relatively more important to U.S. students and relatedness to Kuwaiti students.

## Methodology

### Research design

We employed a survey and cross-cultural research design in the current study. Specifically, we used survey methods to assess perceptions of autonomy, relatedness, and transformative experience along with demographic factors in a U.S. and Kuwaiti sample.

### Ethical statement

Ethical approval for this study was obtained from the Institutional Review Board (IRB) at [Blinded for Review]. All procedures complied with the ethical standards of the American Psychological Association. Informed consent was obtained from all participants prior to their participation. Data collection was anonymous with no identifying information being collected or linked to participants' responses. Data were stored securely on password-protected university servers accessible only to the research team.

Given the cross-cultural nature of the study, particular care was taken to ensure ethical appropriateness across contexts. The survey materials were professionally translated into Arabic and back-translated to ensure conceptual equivalence. As the first author is Kuwaiti and received doctoral training in Educational Psychology at a U.S. institution, cultural and linguistic adaptations were made with attention to both local relevance and international research standards. In Kuwait, additional efforts were made to ensure that consent language was culturally appropriate and accessible. Participants in both countries were informed that their participation was voluntary, that they could withdraw at any time without penalty, and that their responses would remain anonymous and be used for research purposes only. No compensation was provided, and all participation was based on informed choice. These steps were intended to ensure equitable participation and respectful engagement across both cultural settings.

### Participants

Pre-service teachers from one mid-size university in the western U.S. and one large Kuwaiti university comprised the sample. For overall context, the Kuwaiti education system has connections to Dewey's pragmatic educational theory. Jafar (2022) highlighted that the Kuwaiti curriculum makes extensive use of pragmatic educational theory, with an emphasis on linking education to the reality of the learner, making it helpful and practical, and basing learning on the student's life and future.

A total of 408 pre-service teachers completed a study survey administered at the start of the academic year. Of these, 54 participants were dropped because they did not meet selection criteria. Specifically, they reported being in their first year, and thus would have limited experience to reflect on, or failed to report their year in school. The final sample consisted of 353 pre-service teachers: 147 from the U.S. university and 206 from the Kuwaiti university. All participants were 18 years of age or older, and all were enrolled in an undergraduate teacher education program. Participant demographics are displayed in Table 1.

**Table 1:** Demographics.

**Variable**	**U.S. students (*n* = 147)**	**Kuwaiti students (*n* = 206)**
**Gender** ^a^		
Male	18 (12.2%)	8 (3.9%)
Female	123 (83.6%)	198 (96.1%)
Non-binary	6 (4.1%)	
**Race** ^b^		
White	121 (82.3%)	
Hispanic	20 (13.6%)	
Multi-racial	3 (2.0%)	
Other	3 (2.0%)	
**Year in school**		
Year 2	30 (20.4%)	76 (36.9%)
Year 3	69 (46.9%)	75 (36.4%)
Year 4	42 (28.6%)	41 (19.9%)
Year 5	6 (4.1%)	14 (6.8%)
**Age**		
18		7 (3.4%)
19	22 (15.0%)	41 (19.9%)
20	53 (36.1%)	46 (22.3%)
21	37 (25.2%)	35 (17.0%)
22	11 (7.5%)	20 (9.7%)
Above 23	23 (15.6%)	50 (24.3%)
Missing	1 (0.6%)	7 (3.4%)
**Major**		
Early childhood education	18 (12.3%)	6 (2.9%)
Elementary education	62 (42.2%)	
Special education	24 (16.3%)	
Social studies	9 (6.1%)	52 (25.2%)
World languages	10 (6.8%)	81 (39.3%)
Mathematics	4 (2.7%)	31 (15.0%)
Science education	3 (2.0%)	30 (14.6%)
Theater/music/arts	10 (6.8%)	
Student designed major	3 (2.1%)	
Missing	4 (2.7%)	6 (2.9%)

^a^Non-binary is not legally recognized and not culturally accepted in Kuwait. ^b^The core race categories (e.g., White, Black, Hispanic, and etc.) are not culturally recognized in Kuwait.

### Data collection procedure

Data for the study were collected via an online survey between the second and fourth week of the Fall semester. Instructors of pre-service teachers at both universities distributed the study survey link to their students through email or a learning management system (e.g., Canvas). Instructors were encouraged, but not required, to offer those willing to participate in the study a small amount of extra credit. All the questionnaires were translated to Arabic for Kuwaiti students by the first author. After the initial translation, two professional translators reviewed the translation and confirmed the accuracy.

### Measures

#### Transformative Experience Questionnaire

Transformative experience was assessed with a 16-item measure adapted from the Transformative Experience Questionnaire (TEQ; Koskey et al., 2018). Participants were asked to think about their teacher education courses as a whole when responding to the survey. Items assessed the three characteristics of transformative experience: motivated use (e.g., “Outside of school, I use the knowledge I've learned about teaching and learning”), expansion of perception (e.g., “I look for examples of teaching and learning outside of class.”), and experiential value (e.g., “I find that knowledge of teaching and learning make my current, out-of-school experience more meaningful”). In addition, items reflected a continuum ranging from in-class engagement (i.e., applying content during class), to out-of-class engagement (i.e., applying content outside of class), to active out-of-class engagement (i.e., actively seeking opportunities to apply content outside of class). In line with prior use of the TEQ, Rasch analysis (Rasch, 1980) was used to evaluate the measure and develop a composite score. For both samples, TEQ person separation (U.S. = 2.28; Kuwaiti = 2.62) and reliability (U.S. = 0.84; Kuwaiti = 0.87) were strong, indicating distinction between participants in levels of transformative experience and a reliable ordering of participants by levels of transformative experience. TEQ item separation (U.S. = 4.50; Kuwaiti = 3.09) and reliability (U.S. = 0.95; Kuwaiti = 0.91) were also strong.

#### The Learning Climate Questionnaire

Students' perceived sense of autonomy was measured using the Learning Climate Questionnaire (LCQ; Williams and Deci, 1996). The 15-item scale measures students' perceptions of their instructors' support for their autonomy. The scale has one dimension and focuses on students' sense of autonomy inside the classroom (e.g., “I feel that my instructor accepts me”). Two items were dropped as a result of the confirmatory factor analysis (CFA) results (see below). The final 13-item scale had strong reliability for both the U.S. and Kuwaiti samples (α = 0.94 and 0.90, respectively).

#### The University Belonging Questionnaire

Students' perceived sense of relatedness to the university was measured using the University Relatedness Questionnaire (UBQ; Slaten et al., 2018). This 24-item scale measures the extent to which students feel they belong at their university. UBQ has three subscales to measure students' relatedness which are university affiliation (e.g., “I tend to associate myself with my school”), university support and acceptance (e.g., “My university provides opportunities to engage in meaningful activities”), and faculty and staff relations (e.g., “I feel that a faculty/staff member has appreciated me”). Two items were dropped because they were not appropriate to the Kuwaiti context (e.g., item referencing attending university sporting events). Based on the CFA and exploratory factory analysis (EFA) results (see below), one additional item was dropped and we combined the UMQ subscales. The final 23-item scale had strong reliability for both the U.S. and Kuwaiti samples (α = 0.94 and 0.91, respectively).

Confirmatory Factor Analysis (CFA) using Mplus8 was applied to examine the factor structure of the LCQ and UBQ and test whether they separated in the current dataset. As indicators of good fit, we used the guidelines recommended by Byrne (1994) of RMSEA < 0.08, CFI > 0.90, TLI > 0.90, and SRMR < 0.08. The original CFA with all items from both measures and the three UBQ subscales as separate factors had inadequate fit statistics. Consequently, we reviewed items for theoretical fit with the construct and items with lower factors loadings. Two items were dropped from the LCQ. One item was the only reverse coded item, and it loaded poorly on the LCQ factor. A second item appeared to reference belonging more than autonomy (“I feel my instructor cares about me as a person”). Dropping these items improved the model fit. One item was dropped from the UBQ as it had a lower factor loading and targeted perceived similarity in the major instead of sense of belonging in the university as a whole. Dropping this item further improved the model fit. We then used modification indices to further improve the fit of the model.^2^ The final model had good fit apart from the TFI fit being slightly below 0.90: RMSEA = 0.057, CFI = 0.904, TFI = 0.892, SRMR = 0.053. In the final model, all the LCQ items loaded well on the LCQ factor (standardized loadings > 0.597). The UBQ items generally loaded strongly on the respective subscale factors, with three items having standardized loadings below 0.5. To further evaluate the measures, we also conducted exploratory factor analysis (EFA). In the EFA, all the LCQ items loaded on a single factor with no crossloading on any of the UBQ subfactors. The UBQ items all loaded on the three UBQ subfactors with no crossloading on the LCQ factor. However, there were significant crossloadings across the three subfactors. Because of this crossloading in EFA and the needed modifications in the CFA, we choose to combine UBQ subscales. This decision fit with our theoretic model conceptualizing relatedness as a holistic construct (Ryan and Deci, 2017).

### Demographics

The demographic characteristics of the study participants are presented in Table 1. Among U.S. students (*n* = 147), females constituted the majority (83.6%), with males (12.2%) and a minority identifying as non-binary (4.1%). In contrast, Kuwaiti students (*n* = 206) were predominantly female (96.1%), with a small proportion of males (3.9%) and no non-binary students, reflecting cultural and legal norms in Kuwait. Regarding students year in school, U.S. students were primarily in their third year (46.9%), followed by fourth (28.6%), second (20.4%), and fifth year (4.1%). Kuwaiti students were mostly in their second year (36.9%) and third year (36.4%), with smaller proportions in their fourth (19.9%) and fifth year (6.8%).

### Analysis

Hierarchical multiple regression was used to determine if perceived autonomy and relatedness support predicted transformative experience controlling for demographic factors (research question 1), if there was an interaction between the predictors (research question 2), and if there was a three-way interaction between the predictors and students' nationality (research question 3). To help explain the three-way interaction, moderation analyses (Hayes, 2018) were used. The first moderation analysis examined whether the relationship between perceived autonomy support and transformative experiences was moderated by perceived relatedness support, and whether this moderation was invariant across U.S. and Kuwaiti samples. The second analysis was the inverse of the first. It examined whether the relationship between perceived relatedness support and transformative experiences was moderated by perceived autonomy support, and whether this moderation was invariant across U.S. and Kuwaiti samples. The analyses were conducted using conditional process analysis macros (Hayes, 2018) through SPSS software.

## Results

### Descriptive statistics

**Figure 1:**
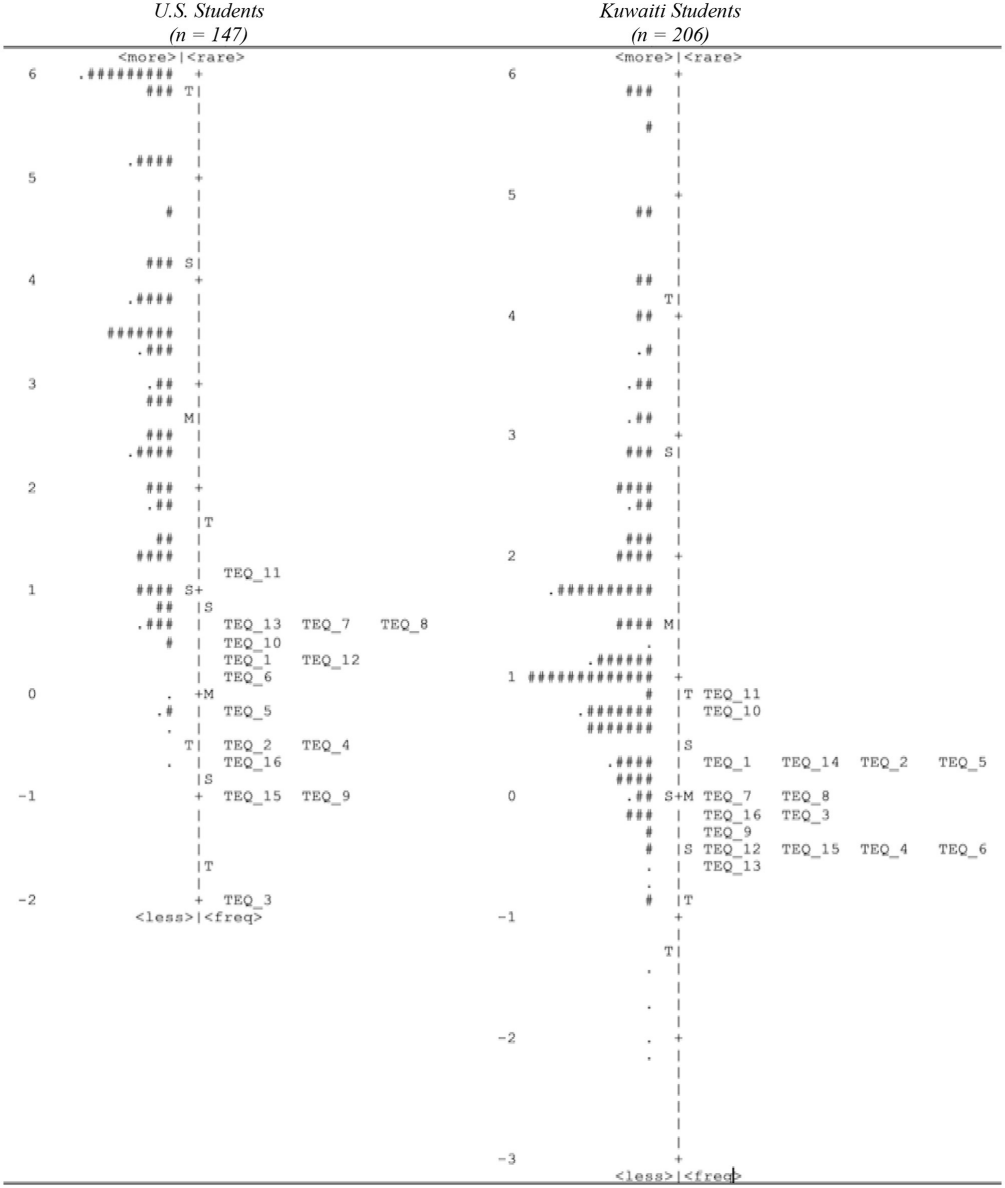
Wright map of Transformative Experience Questionnaire. Each “#” is 2 persons; each “.” is 1 person. TEQ, Transformative Experience Questionnaire item.

Descriptive statistics are displayed in Table 2. Students' perceived sense of autonomy support mean scores for the U.S. and Kuwaiti students were 4.03 and 3.56, respectively. The mean scores illustrate that U.S. students were, on average, agreeing to the autonomy items, while Kuwaiti students were, on average, between neutral and agree. Overall, students in both groups, on average, perceived at least moderate autonomy support. Students' perceived relatedness support mean scores for the U.S. and Kuwaiti students were 3.77 and 3.51, respectively. The mean scores illustrate that both American and Kuwaiti students were, on average, between being neutral and agreeing with the relatedness items, suggesting they had similar moderate perceptions of relatedness support. Students transformative experience mean Rasch scores for the U.S. and Kuwaiti students were 3.04 and 1.62, respectively. Figure 1 provides a Wright Map of Rasch scores for each group. Items are listed on the right side and students on the left. Students are likely to endorse items located below their Rasch score. For both samples, the mean Rasch score, indicated by “M” on the left side of the center line, is located above all the items. This means students were, on average, likely to endorse all the items, even items representative active out-of-school engagement.

**Table 2:** Descriptive statistics.

**Variable**	**U.S. sample**			**Kuwaiti sample**			**Correlations**		
	***N***	***M***	***SD***	***N***	***M***	***SD***	**1**	**2**	**3**
1-Autonomy^a^	147	4.02	0.58	206	3.59	0.65	–	0.54^**^	0.24^**^
2- Relatedness^a^	147	3.84	0.59	204	3.58	0.59	0.45^**^	–	0.41^**^
3-Transformative experience^b^	147	3.04	2.03	206	1.62	1.57	0.39^**^	0.32^**^	–

Correlations for the US and Kuwaiti samples are presented below and above the diagonal, respectively. ^a^Responses were on a five-point Likert scale (1 = Strongly Disagree; 5 = Strongly Agree). ^b^Rasch score < −0.5 = in-class engagement only; 0 = somewhat transformative; > 0.75 = significantly transformative. ^**^Correlation is significant at 0.01 level (two-tailed).

Correlations between variables for U.S. and Kuwaiti students are displayed in Table 2. The predictors (perceived autonomy and relatedness support) showed statistically significant correlations with transformative experience for both groups. In addition, perceived autonomy and relatedness support were statistically significantly correlated for both groups.

### Research question 1

To answer the first research question, hierarchical multiple regression was used to test whether perceived autonomy and relatedness support predicted transformative experience, controlling for demographic factors (see Table 3). In step one, year in school and gender were entered to control for these demographic characteristics. This model was not statistically significant, *F*_(2,348)_ = 1.22, *p* = 0.30, *R*^2^ = 0.007. In step two, perceived autonomy and relatedness support were entered and the model was statistically significant, *F*_(4,346)_ = 25.35, *p* < 0.001. Both perceived autonomy and relatedness support were statistically significant predictors of transformative experience (β = 0.23, *p* < 0.001 and 0.30, *p* < 0.001, respectively) with small-medium effect sizes.^3^ In addition, when perceived autonomy and relatedness support were added to the model, year in school become a statistically significant predictor (β = 0.13, *p* = 0.005) with a small effect size. In addition, the Δ*R*^2^ value of 0.220 associated with this regression model indicates that the addition of perceived autonomy and relatedness support accounts for 22% of the variation in transformative experience scores.

**Table 3:** Hierarchical multiple regression analysis predicting transformative experience.

**Variable**	***B***	**SE *B***	**β**	**R^2^**	**Δ*R*^2^**
Step 1				0.01	0.01
Year	0.18	0.12	0.08		
Gender	−0.02	0.18	−0.01		
Step 2				0.23	0.22^***^
Year	0.29	0.10	0.13^*^		
Gender	−0.11	0.16	0.03		
Autonomy	0.72	0.16	0.24^***^		
Relatedness	0.92	0.18	0.29^***^		
Step 3				0.24	0.02^**^
Year	0.26	0.10	0.12^*^		
Gender	−0.07	0.16	−0.02		
Autonomy	0.72	0.16	0.24^***^		
Relatedness	0.92	0.18	0.29^***^		
Autonomy^*^Relatedness	0.56	0.19	0.14^**^		
Step 4				0.25	0.01^*^
Year	0.27	0.10	0.12^*^		
Gender	−0.07	0.16	−0.02		
Autonomy	0.61	0.17	0.21^***^		
Relatedness	0.81	0.18	0.26^***^		
Autonomy^*^Relatedness	1.17	0.32	0.29^***^		
Autonomy^*^Relatedness^*^Nationality	−0.97	0.41	−0.20^*^		

^*^p < 0.05, ^**^p < 0.01, ^***^p < 0.001. β = 0.01–0.29 = small, 0.30–0.49 = medium, 0.50 or greater = large (Cohen, 1988).

### Research question 2

**Figure 2:**
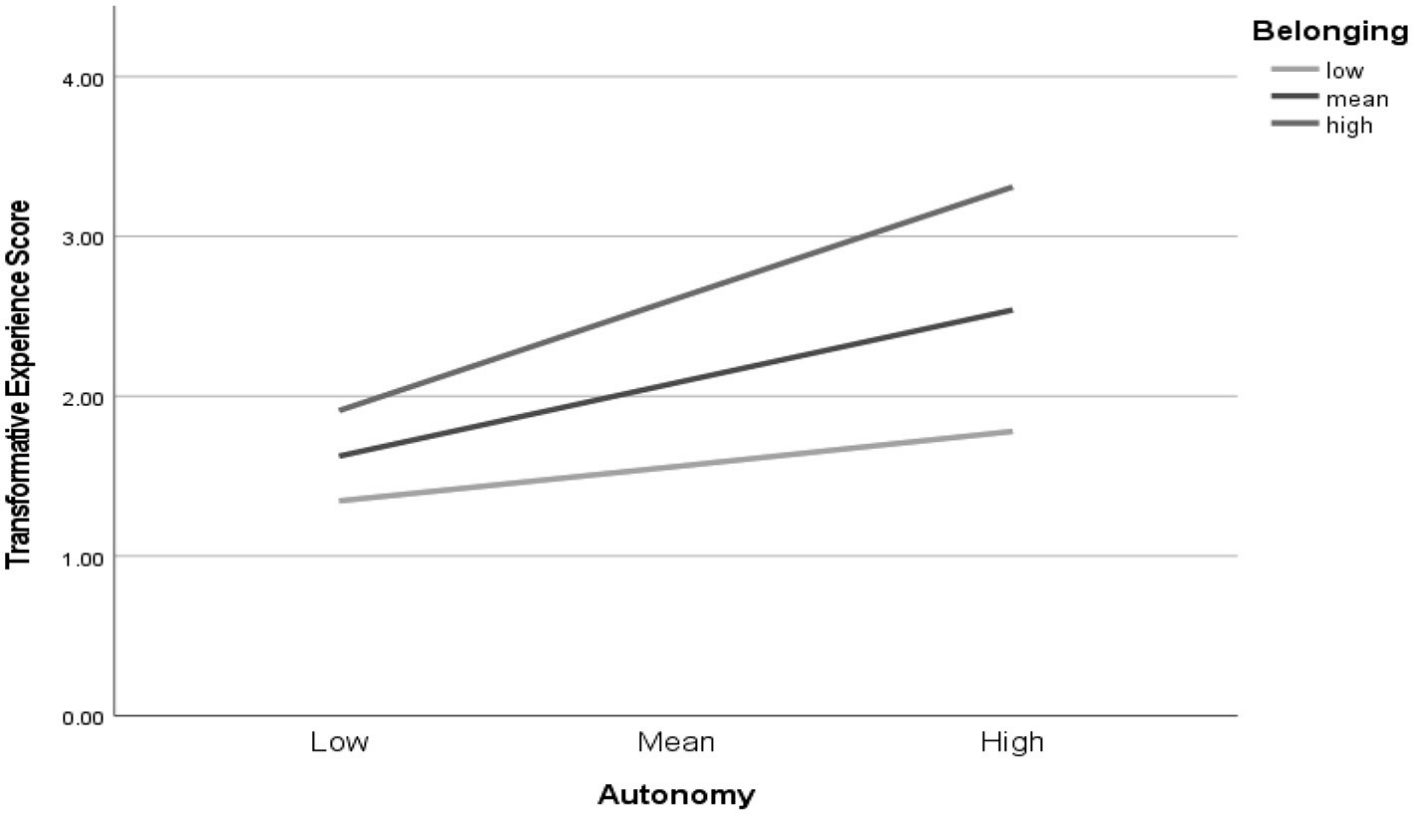
Simple slope equations of the regression of transformative experience on perceived autonomy support at three levels of perceived relatedness support.

At step three of the hierarchical multiple regression, the interaction between perceived autonomy and relatedness support was entered (see Table 3). This interaction was a statistically significant predictor of transformative experience (β = 0.14, *p* < 0.01) with a small effect size and resulted in a statistically significant increase in the amount of variance explained [Δ*R*^2^ = 0.019, *F*_(5,345)_ = 22.48, *p* < 0.001]. Year in school, perceived autonomy support, and perceived relatedness support remained statistically significant predictors in step three. To illustrate the interaction, we created high and low groups with high being 1 standard deviation above the mean and low being 1 standard deviation below the mean. We then plotted these groups and the mean (see Figure 2). The figure illustrates the positive interaction in that an increase in one predictor is associated with an increase in the relation between the other predictor and transformative experience. The nature of these relationships are addressed further in research question 3.

### Research question 3

At step four of the hierarchical multiple regression, the interaction between perceived autonomy support, perceived relatedness support, and nationality was entered (see Table 3). This interaction was a statistically significant predictor of transformative experience (β = −0.20, *p* < 0.05) with a small effect size and resulted in a statistically significant increase in the amount of variance explained [Δ*R*^2^ = 0.012, *F*_(6,344)_ = 19.94, *p* < 0.001]. Year in school, perceived autonomy support, perceived relatedness support, and the autonomy by relatedness interaction remained statistically significant predictors in step four.

**Figure 3:**
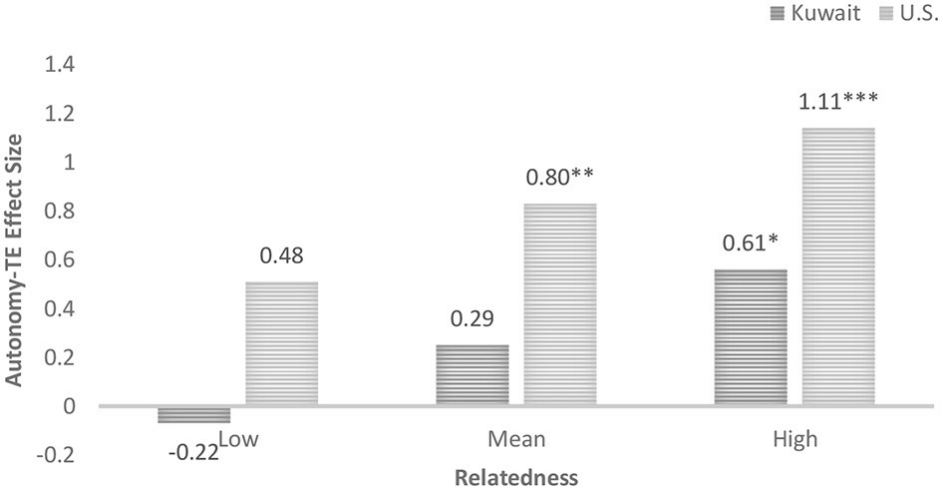
Effect size of the relation between perceived autonomy support and transformative experience (TE) at different levels of perceived relatedness support by nationality. *p < 0.05, **p < 0.01, ***p < 0.001.

To explain the three-way interaction, moderation analyses (Hayes, 2018) were run. Moderation analysis showed that when perceived relatedness support is low, 1 standard deviation below the mean, there is a non-significant positive relationship between perceived autonomy support and transformative experience for U.S. students, *b* = 0.49, 95% CI [−0.11, 1.08], *t* = 1.61, *p* = 0.11, and a non-significant negative relationship for Kuwaiti students, *b* = −0.022, 95% CI [−0.42, 0.38], *t* = −0.11, *p* = 0.91. See Figure 3 which illustrates the effect size of the relationship between perceived autonomy support and transformative experience at different levels of perceived relatedness support. However, when perceived relatedness support is at the mean value, the positive relationship between perceived autonomy support and transformative experience is significant for U.S. students, *b* = 0.80, 95% CI [0.30, 1.3], *t* = 3.18, *p* < 0.01, but not for Kuwaiti students, *b* = 0.29, 95% CI [−0.10, 0.69], *t* = 1.45, *p* = 0.15. When perceived relatedness support is 1 standard deviation above the mean, there is a significant positive relationship between perceived autonomy support and transformative experience for both U.S. students at *b* = 1.12, 95% CI [0.62, 1.61], *t* = 4.44, *p* < 0.001 and Kuwaiti students at *b* = 0.61, 95% CI [0.09, 1.12], *t* = 2.34, *p* = 0.02.

**Figure 4:**
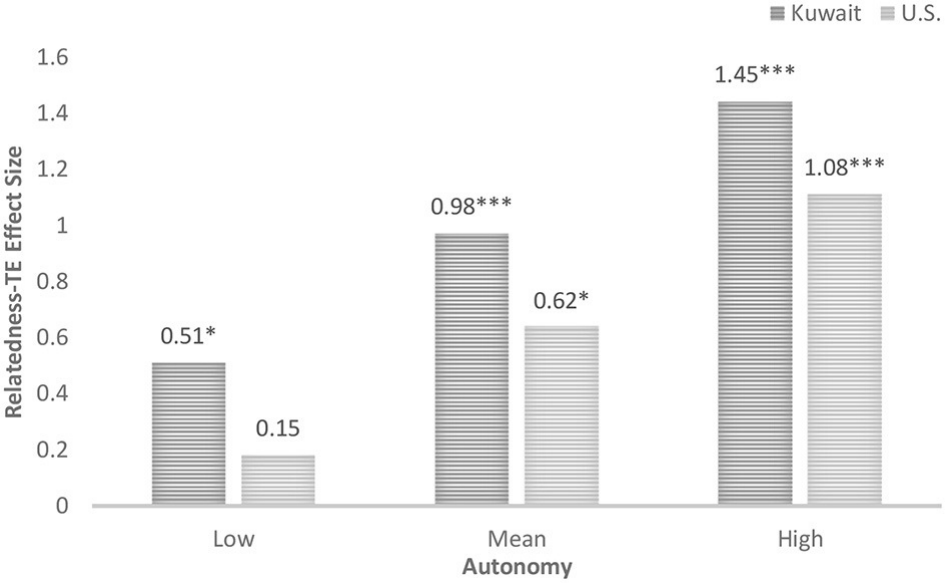
Effect size of the relation between perceived relatedness support and transformative experience (TE) at different levels of perceived autonomy support by nationality. *p < 0.05, ***p < 0.001.

To further illustrate the interaction the second moderation analysis was ran with perceived autonomy support as the moderator. The moderation analysis showed that when perceived autonomy support is low, 1 standard deviation below the mean, there was a non-significant positive relationship between perceived relatedness support and transformative experience for U.S. students, *b* = 0.15, 95% CI [−0.48, 0.77], *t* = 0.46, *p* = 0.65, but a significant positive relationship for Kuwaiti students, *b* = 0.51, 95% CI [0.05, 0.97], *t* = 2.14, *p* = 0.03 (see Figure 4). However, when perceived autonomy support was at the mean value, the relationship between perceived relatedness support and transformative experience was significant for both U.S. students, *b* = 0.62, 95% CI [0.12, 1.11], *t* = 2.44, *p* = 0.015, and Kuwaiti students*, b* = 0.98, 95% CI [0.54, 1.41], *t* = 4.48, *p* < 0.001. Additionally, when perceived autonomy support was high, 1 standard deviation above the mean, there was a significant positive relationship between perceived relatedness support and transformative experience for both U.S. students, *b* = 1.08, 95% CI [0.61, 1.56], *t* = 4.45, *p* < 0.001, and Kuwaiti students *b* = 1.45, 95% CI [0.9, 1.98], *t* = 5.33, *p* < 0.001.

## Discussion

In line with our first hypothesis, controlling for gender and year in school, we found that perceived autonomy and relatedness support were positively associated with transformative experience with a small to moderate effect size. These results align with previous research identifying an association between students' perceived autonomy support and other engagement outcomes. Autonomy has been empirically established as a core factor explaining engagement in autonomous motivation, particularly intrinsic motivation (Ryan and Deci, 2017; Wang et al., 2025). In addition, sense of autonomy has been positively associated with mastery goals (Benita and Matos, 2021), interest (Gillet et al., 2012), positive task values (Patall et al., 2013), and overall engagement (Benita and Matos, 2021; Gutiérrez and Tomás, 2019; Hospel and Galand, 2016; Jang et al., 2010; Reeve et al., 2004). These findings also echo Pugh et al.'s (2010) work showing mastery goal orientation as a mediator between science identity and transformative experience, reinforcing the broader motivational processes underpinning transformative engagement. The results are also consistent with prior research finding a link between perceived relatedness support and engagement outcomes. As with autonomy, relatedness has been empirically established as a core factor related to engagement in intrinsic motivation (Ryan and Deci, 2017). Relatedness is also positively associated with academic self-efficacy (Freeman et al., 2007), positive task values (Freeman et al., 2007), and engagement in general (Finn and Zimmer, 2012; Gillen-O'Neel, 2019; Gopalan and Brady, 2020; Wilson et al., 2015; Zumbrunn et al., 2014). Additionally, prior studies like Heddy et al. (2023) demonstrated domain interest as a predictor of transformative experience, suggesting that the motivational landscape includes both personal and relational dimensions.

In line with our second hypothesis, controlling for gender and year in school, we found a significant interaction between perceived autonomy and relatedness support in predicting transformative experience, with a small effect size. This result is in line with the theorizing of Deci and Ryan (2000) and Kluwer et al. (2020) who suggest that autonomy and relatedness play a complementary rather than antagonistic role in the human psyche. That is, autonomy does not imply isolation, but rather refers to having the ability to choose while still feeling connected to others.

In agreement with our third hypothesis, controlling for gender and year in school, we found a significant three-way interaction between perceived autonomy support, perceived relatedness support, and nationality, with a small effect size. In line with our follow-up hypotheses, we found perceived autonomy and relatedness support to each be associated with transformative experience for both the U.S. and Kuwaiti students. In addition, we also found variation in relationship strength and moderation effects by nationality, suggesting that the salience and functioning of perceived autonomy and relatedness support varies by culture. However, caution is warranted when interpreting these cultural differences. The study's cross-sectional design and reliance on self-report measures limit the ability to make strong claims about cultural differences. Observed differences may reflect reporting styles, unmeasured contextual factors, or sampling variations rather than true cultural mechanisms.

The strength of the relationship between perceived autonomy support and transformative experience was stronger for U.S. students compared to Kuwaiti students. This individual difference was consistent across different levels of relatedness (see Figure 3). Further, we found that a certain baseline or threshold level of perceived relatedness support appeared to be associated with a stronger relationship between perceived autonomy support and transformative experience. However, this threshold was lower for U.S. students. U.S. students exhibited an association between perceived autonomy support and transformative experience even when reporting an average level of perceived relatedness support. In contrast, Kuwaiti students exhibited a significant association only when reporting high levels of perceived relatedness support. For both groups, the association between perceived autonomy support and transformative experience became more pronounced as levels of perceived relatedness support increased. We found similar patterns when examining how perceived autonomy support moderated the association between perceived relatedness support and transformative experience.

The association between perceived relatedness support and transformative experience was stronger for Kuwaiti students compared to U.S. students across all levels of autonomy (see Figure 4). In addition, we found that, for U.S. students but not Kuwaiti students, a baseline level of perceived autonomy support appeared to be associated with the emergence of a significant relationship between perceived relatedness support and transformative experience. U.S. students exhibited a significant association between perceived relatedness support and transformative experience when they reported at least an average level of perceived autonomy support. Kuwaiti students exhibited a significant association even when perceived autonomy support was low. For both groups, the association between perceived relatedness support and transformative experience became more pronounced as levels of autonomy increased. These findings must also be interpreted in light of several methodological limitations. Self-report measures may be influenced by social desirability or cultural response biases. Additionally, because this study employed a convenience sample and a cross-sectional design, causal inferences and generalizations beyond the sampled populations should be made cautiously.

Overall, the cross-cultural results are consistent with the assertion made by Ryan and Deci (2020) regarding both the universal importance of basic psychological needs and the culture-specific variation in the salience, meaning, and dynamics of these needs. Perceived autonomy and relatedness support were found to be significantly associated with transformative experience for both U.S. and Kuwaiti students, suggesting the universal role of these needs and contrasting claims by some researchers (e.g., Markus and Kitayama, 2003) that autonomy is not important in a collectivistic culture like Kuwait. However, the results also revealed that perceived autonomy support was relatively more important for U.S. students than Kuwaiti students, and perceived relatedness support was relatively more important for Kuwaiti students than U.S. students. These findings are consistent with the claim made by Magson et al. (2024) that all self-determination theory psychological needs are important for people across cultures, but that specific needs exert greater influence on outcomes depending on cultural context. Our findings also revealed differences in the strength and interaction of autonomy and relatedness support between U.S. and Kuwaiti students; thus supporting the argument made by Heine et al. (1999) about the critical role of relatedness in collectivist cultures. Moreover, these patterns mirror the gender-specific differences in transformative experience found by Pugh et al. (2021), where demographic and contextual factors shaped how psychological needs translated into educational outcomes.

However, we caution against interpreting these differences solely through an individualism–collectivism lens, which risks oversimplifying cultural influences. Autonomy and relatedness are universal psychological needs (Ryan and Deci, 2020), but their expression varies with sociocultural norms and institutional contexts (Vignoles et al., 2016). Rather than directly mapping autonomy to individualism or relatedness to collectivism, we adopt a culturally sensitive approach that recognizes both the universal importance and context-specific expressions of these needs. This perspective aligns with Ryan and Deci's (2020) view that psychological needs are essential across cultures but are shaped by local values and practices.

Finally, although not a focus of the current research, we found the control variable, year in school, to be a small but statistically significant predictor of transformative experience when the other predictors were entered in the model. Thus, accounting for autonomy and relatedness, students more senior in their teacher education program were more likely to report higher levels of transformative experience.

### Implications

Although the present results are correlational and do not establish causality, they align with prior research highlighting connections between perceived autonomy and relatedness support and engagement outcomes (e.g., Gutiérrez and Tomás, 2019; Hospel and Galand, 2016; Gillen-O'Neel, 2019; Gopalan and Brady, 2020). These findings imply that teachers may foster transformative experience by supporting students' autonomy and relatedness needs. In particular, emphasizing autonomy support may be especially impactful in U.S. educational contexts, while emphasizing relatedness support may be particularly important in Kuwaiti settings. Instructors can provide autonomy support in teacher education courses by assisting students in pursuing self-selected goals, making them feel valued and welcomed, offering opportunities for student voice, praising signs of progress and mastery, and supporting students' work (Hensley et al., 2021; a, ngReeve and Jang2006). Additionally, to support students' relatedness needs, instructors in teacher education courses may use cooperative learning, assist students in developing prosocial behavior, and maintain positive connections with students (Bergin, 2018; Ryan and Deci, 2017). Tailoring educational strategies to the cultural context may enhance the likelihood of promoting transformative learning experiences across diverse student populations. However, researchers need to investigate this possibility with experimental research methods.

### Limitations

This study has several limitations that should be considered. Because the sample was a convenience sample, it is difficult to assume that the sample is representative of the population, which impacts the generalizability of the results. Second, the study relied exclusively on self-report measures, which may introduce biases such as social desirability and common method variance. Third, although validated scales were used to measure perceived autonomy and relatedness support, it is possible that cultural differences in how these constructs are interpreted may have affected the comparability of responses across groups, raising concerns about cross-cultural construct validity. Finally, given the correlational nature of the study, conclusions about causal relationships between psychological needs and transformative experiences cannot be drawn.

### Future research

Future research should seek to replicate this study in different populations to assess the generalizability of the findings across diverse educational and cultural contexts. Additionally, future work could examine whether students' sense of competence—another basic psychological need identified by Ryan and Deci (2017)—is significantly associated with transformative experience. Investigating factors that influence the strength of the relationships between perceived autonomy and relatedness support and transformative experience, such as contextual or individual difference variables, would also be valuable. Finally, future research employing longitudinal designs would help clarify the directional and causal pathways among these constructs. Likewise, autonomy and relatedness intervention studies could provide evidence for these factors fostering transformative experience.

## Conclusion

The purpose of this study was to better understand how perceived autonomy and relatedness support are associated with transformative experience, and how these factors may interact in relation to transformative experience. Additionally, the study examined whether these relationships differed based on students' nationality. The findings indicated that both perceived autonomy and relatedness support were significantly associated with transformative experience, and that these factors interacted positively. Perceived autonomy and relatedness support were important in both the U.S. and Kuwaiti samples, suggesting the universal importance of these basic psychological needs. However, moderation analyses revealed culturally specific patterns, with perceived autonomy support playing a relatively stronger role for U.S. students, and perceived relatedness support playing a relatively stronger role for Kuwaiti students. These results contribute to a growing body of research highlighting both the universal and culturally nuanced roles of psychological needs in shaping meaningful educational experiences.

## Data Availability

The raw data supporting the conclusions of this article will be made available by the authors, without undue reservation.

## Footnotes

^1^Researchers distinguish between basic psychological needs, perceived need satisfaction, and perceived need support (Vansteenkiste and Ryan, 2013). Basic psychological needs are an individual's “actual” psychological needs. Perceived need satisfaction is an individual's perception of the extent to which their psychological needs are met (Wilson et al., 2006), while perceived need support is an individual's perception of the degree to which their environment supports their psychological needs (Deci et al., 2001).

^2^Errors were allowed to correlate between some items in the same factor and between some items across the UBQ subscales; two university affiliation UBQ subscale items were allowed to also load on the UBQ faculty and staff relations subscale.

^3^Cohen (1988), guidelines for interpretingβ: 0.01–0.29 = small, 0.30–0.49 = medium, 0.50 or greater = large.
